# Motion compensation with a scanned ion beam: a technical feasibility study

**DOI:** 10.1186/1748-717X-3-34

**Published:** 2008-10-14

**Authors:** Sven Oliver Grözinger, Christoph Bert, Thomas Haberer, Gerhard Kraft, Eike Rietzel

**Affiliations:** 1 Gesellschaft für Schwerionenforschung (GSI), Abteilung Biophysik, Planckstraße 1, 64291 Darmstadt, Germany

## Abstract

**Background:**

Intrafractional motion results in local over- and under-dosage in particle therapy with a scanned beam. Scanned beam delivery offers the possibility to compensate target motion by tracking with the treatment beam.

**Methods:**

Lateral motion components were compensated directly with the beam scanning system by adapting nominal beam positions according to the target motion. Longitudinal motion compensation to mitigate motion induced range changes was performed with a dedicated wedge system that adjusts effective particle energies at isocenter.

**Results:**

Lateral compensation performance was better than 1% for a homogeneous dose distribution when comparing irradiations of a stationary radiographic film and a moving film using motion compensation. The accuracy of longitudinal range compensation was well below 1 mm.

**Conclusion:**

Motion compensation with scanned particle beams is technically feasible with high precision.

## Background

In conformal radiotherapy, geometric margins are commonly used to account for intra-fractional target motion [1,2]. These margins inevitably lead to inclusion of healthy tissue in the treated volume. In intensity modulated radiotherapy, additional motion effects arise due to so called interplay effects [3-5]. Treatments are delivered in small partial doses that only result in adequate total dosage if they match as intended. In anatomy's eye view, target motion leads to relative displacement of partial dose depositions and therefore results in local over- and under-dosage.

In a pilot project at Gesellschaft für Schwerionenforschung (GSI) [6-9], approximately 400 patients have been treated with scanned carbon ion beams with the rasterscan system [10]. For raster scanning, the target volume is divided in slices corresponding to equal ion energies. Irradiations are performed slice-by-slice. The required particle energy is requested from the synchrotron for each slice. Within each slice, a narrow pencil beam is scanned on a virtual raster grid. To achieve the desired dose distribution, the number of particles is optimized for each raster position during treatment planning including biological effects [11-16]. The scanning progress is intensity controlled. The carbon ion pencil beam is directed to the next raster position by a magnetic deflection system as soon as the planned number of particles has been deposited. After all points within a slice have been irradiated, the beam is aborted and the next energy level is requested from the accelerator. To date, only patients with tumors that are not subject to intra-fractional motion have been treated [7,17-19]. For treatments with scanned particle beams, target motion would inevitably lead to local over- and under-dosage due to the relative lateral motion between pencil beam positions as well as possible motion induced changes in radiological depths.

To treat moving targets, while maintaining the conformity between target and treated volume as well as avoiding local over- and under-dosage, we are investigating and developing a system to adapt 3D pencil beam positions to actual target positions in real time. Initially, simulation studies were performed to investigate the potential of target tracking with a scanned ion beam [4,20]. In beam's eye view, lateral motion adaptation of pencil beam positions is feasible by applying offsets to the raster scanner settings. Real time energy adaptation to compensate changes in radiological depth with the synchrotron directly is not (yet) possible. Therefore online adaptation of particle ranges has to be performed with an additional, dedicated energy modulation system. One of the possibilities is to use a dedicated absorber wedge system [21].

Prototype systems for lateral as well as longitudinal target tracking with a scanned ion beam have been developed. Experimental results are presented to demonstrate the feasibility of target tracking with a scanned ion beam and to show the performance of the individual prototype tracking sub-systems.

## Methods

### Simulation of target motion

Lateral target motion orthogonal to the beam direction was achieved with a three-axes positioning table. A radiographic film was mounted on the table as detector. The motion was sinusoidal with a period of ~10 s and amplitudes of ± 15 mm in horizontal as well as vertical direction. No external motion monitoring device was used, instead table motion was continuously measured with encoders. Target displacements were evaluated from encoder data and sent directly to the therapy control system (TCS) for beam adaptation during irradiations.

To simulate motion induced variations in particle range, different particle energies were requested from the synchrotron. In a first experiment, three different particle energies were requested from the accelerator repeatedly in fixed order. The energy modulation system was used to adapt the effective particle energy at isocenter to the middle energy. In a second experiment, six different particle energies were requested in mixed order to test the functionality of the system for variable and alternating energy modulations. The maximum difference in energy corresponded to a water equivalent range difference of 27 mm. Again, the energy modulation system was used to adapt the effective particle energy to a single range.

### 3D online motion compensation

#### Lateral motion compensation

The raster scanning process is controlled by the TCS. Beam position as well as delivered number of particles are monitored in intervals of ~150 μs and ~10 μs respectively. The standard TCS can adjust small deviations of the actual beam position via a fast feedback loop. Whenever the beam position has been measured, possible deviations are fed back to the control of the scanning magnets to correct the beam position to the nominal position. Typically, deviations are within ± 0.5 mm and corrected after each measurement cycle. The irradiation time for an individual raster point is typically in the order of 5–10 ms.

Several processes are running simultaneously in the TCS including monitoring of the beam intensity, the beam position, and the raster scanner magnet settings. The individual processes communicate via a control loop as well as shared memory. For motion compensation, adaptation of lateral pencil beam positions was implemented by dynamically changing the nominal values of the beam positions in shared memory. As soon as the nominal values have been changed, the feedback loop adjusts the beam position accordingly. A dedicated, additional process running on the TCS receives displacement vectors and then changes the nominal beam positions in shared memory accordingly. In order to avoid hardware changes within the TCS for the prototype setup, a standard network connection (100 Hz) was used to transmit displacement vectors to the TCS. The actual displacement vector is added to the stationary nominal raster point position to compute the new, dynamic nominal position.

#### Longitudinal motion compensation

**Figure 1:**
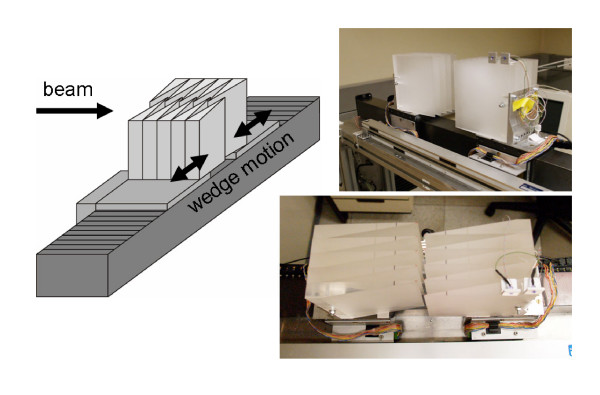
**Energy modulation system**. Two opposing wedge shaped absorbers are mounted on linear motors between beam exit window and isocenter to continuously adjust the effective energy; left: schematic drawing, right: photographs in oblique side view (upper) and top view (lower).

To perform motion compensation in longitudinal direction, the energy of individual pencil beams has to be adjusted in quasi real time. Because fast active energy variation with the accelerator is not possible, a passive energy modulation system was developed and installed between beam exit window and isocenter [21]. The system consists of two opposing lucite wedge absorbers that are mounted on linear motor drives orthogonal to the beam direction (figure 1). By moving the wedges apart (together) with the linear motors, the thickness of absorber material in the beam path can be decreased (increased) to adapt the effective beam range at isocenter fast and continuously. The system has an active compensation area of 120 × 150 mm^2^. The absorber wedges were designed to provide homogeneous range adaptation within the active area by adequate overlap. If the treatment field exceeds the dimension of the active area in the horizontal direction of wedge motion both wedges can be moved synchronously to provide adequate range adaptation. The total wedge thickness of the prototype system corresponds to a maximum water equivalent range variation of ± 49.4 mm which should exceed the maximum clinically required range adaptation.

### Measurement and analysis of dose distributions

Different detectors were used to measure dose distributions: planar radiographic films for lateral 2D dose distributions and a range telescope for longitudinal 1D depth dose distributions [22].

Radiographic films (Kodak X-Omat V) were developed with a Kodak M35 processing machine. The films were digitalized with a Kodak LS75 laser densitometer and the FIPS Plus software for film dosimetry (PTW Freiburg) with a spatial resolution of 1 mm. Based on the film responses, absorbed doses were calculated according to Bathelt et al [23] and Spielberger at al [24]. Simple treatment plans were optimized to deliver homogeneous, quadratic dose distributions as well as line patterns. Geometric properties of motion compensation were assessed from the line patterns. For quadratic fields, the homogeneity index H was computed to compare dose distributions quantitatively:

(1) $$H=1-\frac{1}{\bar{D}}\sqrt{\frac{\sum_{i}{\left(D_{i}-\bar{D}\right)}^{2}}{N-1}}$$

with *D*_*i *_dose to each individual pixel, *N *number of pixels within the target area, and $\bar{D}$ mean dose within the target area.

The range telescope was used to measure depth dose distributions, so called Bragg peaks. The telescope consists of two parallel plate ionization chambers in front of and behind a water tank of variable thickness [22,25,26]. During the measurements, the thickness of the water tank was increased in steps of 50 μm.

## Results

### Lateral motion compensation

**Figure 2:**
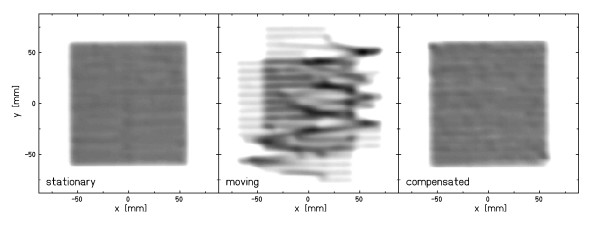
**Lateral motion compensation**. Dose distributions measured with radiographic films: stationary, moving, and moving using lateral motion compensation. Homogeneity indices were 0.969, 0.655, and 0.963 respectively.

Figure 2 shows film responses for a quadratic, homogeneous field. Under motion, marked local over- as well as under-dosage are apparent and relevant dose is deposited outside of the target area. Lateral motion compensation restored the dose distribution on the moving film. In comparison to the reference dose distribution, only small differences within the irradiated area are visible. Homogeneity indices were 0.969, 0.655, and 0.963 for the dose distributions measured under stationary, moving, and motion compensated conditions respectively.

**Figure 3:**
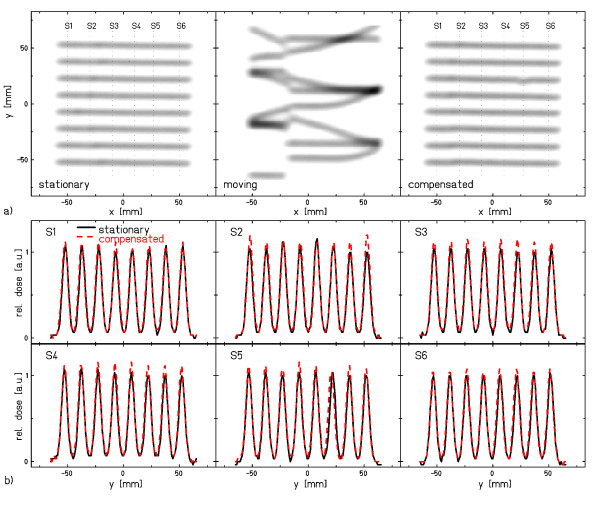
**Geometrical performance of lateral motion compensation**. a) Line patterns irradiated on radiographic films: stationary, moving, and moving using lateral motion compensation. b) Line profiles of the particle fluences in vertical direction at the positions indicated on the film measurements.

Film responses for line patterns are shown in figure 3a. In contrast to the regular, parallel lines on the stationary film, heavily distorted patterns were measured with the moving film. Motion compensation successfully restored the line patterns. A small residual motion artifact is present in the third line from top which was attributed to a sporadic communication delay between motion monitoring and compensation due to communication via a standard network connection. Figure 3b presents line profiles of the film responses for stationary and motion compensated measurements. Positional differences of the lines were on average 0.2 ± 0.2 mm. A maximum deviation of 1.6 mm was observed in the region of the residual motion artifact (figure 3b; S5). Differences in relative dose between the two experiments are within the precision of film measurements.

### Longitudinal motion compensation

**Figure 4:**
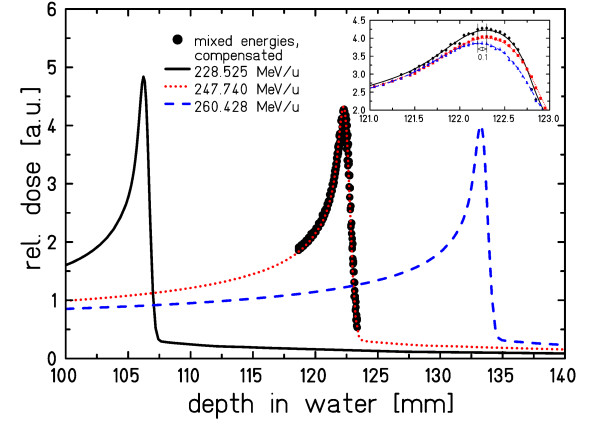
**Longitudinal motion compensation**. Bragg peaks measured for three different energies and obtained by longitudinal motion compensation to the central energy. The inlay shows the sub-millimeter accuracy of longitudinal motion compensation.

The precision of longitudinal motion compensation is presented in figure 4. During irradiation, three different energy levels were adapted to the middle energy using the energy modulation system. The inlay shows that the difference to an individually measured depth dose distribution at the mean energy is ~0.1 mm.

**Figure 5:**
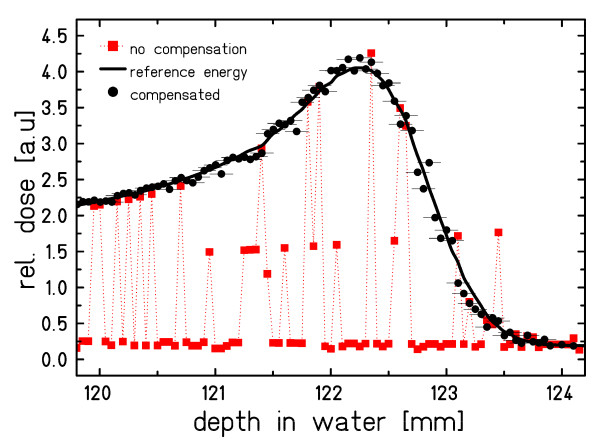
**Performance of longitudinal motion compensation for mixed energies**. Longitudinal motion compensation for 6 different energies requested in random order over a range of 27 mm water-equivalence. Horizontal bars indicate the compensation precision of 0.1 mm water-equivalent.

The performance of energy adaptation for 6 different energy levels requested in random order from the accelerator is shown in figure 5. The energy modulation system successfully restored a single, effective particle energy at isocenter. Fluctuations around the reference depth dose distribution of ~2.5% on average (normalized to the Bragg peak) are mainly attributed to residual calibration uncertainties of the energy modulation system.

## Discussion

The results of our feasibility study demonstrate that motion compensation with scanned particle beams is feasible with high precision. Lateral as well as longitudinal compensation were successfully performed during irradiations. In a next step, both motion compensation sub-systems have to be integrated in the therapy control system. Especially replacing standard network connections to transmit compensation parameters should improve the reliability of the system. Furthermore, hardware improvements of the energy modulation system for longitudinal range compensation should be investigated, and implementation of motion monitoring has to be developed.

Re-design of the wedge system for fast longitudinal motion compensation is advisable since the thickness of the wedges can most likely be reduced to the compensation range required for patient treatments in order to reduce lateral scattering as well as fragmentation of the primary particle beam [27-29]. Furthermore, the active area of the wedge system (120 × 150 mm^2^) does currently not match the treatment area of the scanning system (200 × 200 mm^2^). The wedge size thus has to be increased at least in vertical direction. In contrast, the horizontal dimension of the active area does not necessarily have to match the scan area. If the center of mass of the wedges follows the left-right motion of the ion beam during raster scanning, an active area that is smaller than the maximum treatment area is sufficient. However, less wedge motion and therefore reduced system performance is required if the active area is sufficiently large to cover the complete scanning area. Detailed requirements on the compensation speed have to be derived from simulation studies, for example based on 4D computed tomography data [30-32].

Another problem of motion tracking that has not yet been solved adequately is precise monitoring of target motion. To date, several different methods have been reported in the literature. Currently, the most promising technique seems to be fluoroscopic motion detection because target motion is imaged directly [33-38]. Other techniques that monitor external surface motion have to be evaluated regarding the accuracy to derive target positions [39-46]. Since the particle range and thus the Bragg peak position are influenced by target motion and currently no motion monitoring system exists to determine changes in water-equivalent range a link to 4D treatment planning is required [47,48]. Motion states from 4DCT which are used to determine range changes could be detected by motion monitoring. Compensation vectors are then calculated during treatment planning and applied according to detected motion states. In case of motion irregularities or unknown motion states the treatment can be paused until the patient is back to normal breathing.

## Conclusion

The results of our study demonstrate the high precision that is technically feasible for motion tracking with scanned particle beams. Lateral motion compensation restored homogeneous dose distributions delivered to moving targets. Differences in dose uniformity between irradiation of a stationary radiographic film and a moving film using motion compensation were below 1%. Longitudinal compensation precision was well below 1 mm.

## Competing interests

SOG and ER are now employed by Siemens Healthcare. Research was performed while both were employed by GSI.

## Authors' contributions

All authors contributed to the design of the prototype system and the conceptual design of the study. Furthermore, SOG performed measurements, analyzed data, and drafted the manuscript. CB and ER supported measurements, analyzed data, and revised the manuscript. TH and GK improved the conceptual design and revised the manuscript. All authors read and approved the final manuscript.

## References

[B1] ICRU (1993). Report 50.

[B2] ICRU (1999). Report 62.

[B3] Phillips MH, Pedroni E, Blattmann H, Boehringer T, Coray A, Scheib S (1992). Effects of respiratory motion on dose uniformity with a charged particle scanning method. Phys Med Biol.

[B4] Grozinger SO, Rietzel E, Li Q, Bert C, Haberer T, Kraft G (2006). Simulations to design an online motion compensation system for scanned particle beams. Phys Med Biol.

[B5] Bert C, Grözinger SO, Rietzel E (2008). Quantification of interplay effects of scanned particle beams and moving targets. Phys Med Biol.

[B6] Kraft G (2000). Tumor Therapy with Heavy Charged Particles. Prog Part Nucl Phys.

[B7] Debus J, Haberer T, Schulz-Ertner D, Jakel O, Wenz F, Enghardt W, Schlegel W, Kraft G, Wannenmacher M (2000). Carbon ion irradiation of skull base tumors at GSI. First clinical results and future perspectives. Strahlenther Onkol.

[B8] Schulz-Ertner D, Jakel O, Schlegel W (2006). Radiation therapy with charged particles. Semin Radiat Oncol.

[B9] Schulz-Ertner D, Tsujii H (2007). Particle Radiation Therapy Using Proton and Heavier Ion Beams. J Clin Oncol.

[B10] Haberer T, Becher W, Schardt D, Kraft G (1993). Magnetic scanning system for heavy ion therapy. Nucl Instrum Meth A.

[B11] Krämer M, Scholz M (2000). Treatment planning for heavy-ion radiotherapy: calculation and optimization of biologically effective dose. Phys Med Biol.

[B12] Krämer M, Jäkel O, Haberer T, Kraft G, Schardt D, Weber U (2000). Treatment planning for heavy-ion radiotherapy: physical beam model and dose optimization. Phys Med Biol.

[B13] Krämer M, Scholz M (2006). Rapid calculation of biological effects in ion radiotherapy. Phys Med Biol.

[B14] Scholz M, Kraft G (1994). Calculation of heavy ion inactivation probabilities based on track structure, x ray sensitivity and target size. Radiat Prot Dosim.

[B15] Scholz M, Kellerer AM, Kraft-Weyrather W, Kraft G (1997). Computation of cell survival in heavy ion beams for therapy. The model and its approximation. Radiat Environ Biophys.

[B16] Jäkel O, Krämer M, Karger CP, Debus J (2001). Treatment planning for heavy ion radiotherapy: clinical implementation and application. Phys Med Biol.

[B17] Schulz-Ertner D, Nikoghosyan A, Thilmann C, Haberer T, Jäkel O, Karger C, Kraft G, Wannenmacher M, Debus J (2004). Results of carbon ion radiotherapy in 152 patients. Int J Radiat Oncol.

[B18] Schulz-Ertner D, Nikoghosyan A, Hof H, Didinger B, Combs SE, Jakel O, Karger CP, Edler L, Debus J (2007). Carbon ion radiotherapy of skull base chondrosarcomas. Int J Radiat Oncol Biol Phys.

[B19] Nikoghosyan A, Schulz-Ertner D, Didinger B, Jakel O, Zuna I, Hoss A, Wannenmacher M, Debus J (2004). Evaluation of therapeutic potential of heavy ion therapy for patients with locally advanced prostate cancer. Int J Radiat Oncol Biol Phys.

[B20] Li Q, Grözinger SO, Haberer T, Rietzel E, Kraft G (2004). Online compensation of target motion with scanned particle beams: simulation environment. Phys Med Biol.

[B21] Weber U, Becher W, Kraft G (2000). Depth scanning for a conformal ion beam treatment of deep seated tumours. Phys Med Biol.

[B22] Sihver L, Schardt D, Kanai T (1998). Depth-dose distributions of high-energy carbon, oxygen and neon beams in water. Jpn J Med Phys.

[B23] Bathelt B (2000). Filmdosimetrie in der Schwerionen-Tumortherapie: 3-dimensionale Dosisverifikation in gemischten Teilchenstrahlfeldern.

[B24] Spielberger B, Scholz M, Krämer M, Kraft G (2001). Experimental investigations of the response of films to heavy-ion irradiation. Phys Med Biol.

[B25] Schardt D, Stelzer H, Junk H, Arndt U (1993). Bragg curve measurements with ionisation chambers. Grundinger, U. 336. Darmstadt, Gesellschaft für Schwerionenforschung mbH GSI Scientific Report 1992.

[B26] Rietzel E, Schardt D, Haberer T (2007). Range accuracy in carbon ion treatment planning based on CT-calibration with real tissue samples. Radiat Oncol.

[B27] Schardt D, Schall I, Geissel H, Irnich H, Kraft G, Magel A, Mohar MF, Munzenberg G, Nickel F, Scheidenberger C, Schwab W, Sihver L (1996). Nuclear fragmentation of high-energy heavy-ion beams in water. Adv Space Res.

[B28] Schall I, Schardt D, Geissel H, Irnich H, Kankeleit E, Kraft G, Magel A, Mohar MF, Mnnzenberg G, Nickel F, Scheidenberger C, Schwab W (1996). Charge-changing nuclear reactions of relativistic light-ion beams (5 <= Z <= 10) passing through thick absorbers. Nucl Instrum Meth B.

[B29] Gunzert-Marx K, Iwase H, Schardt D, Simon RS (2008). Secondary beam fragments produced by 200 MeVu(-1) C-12 ions in water and their dose contributions in carbon ion radiotherapy. New Journal of Physics.

[B30] Ford EC, Mageras GS, Yorke E, Ling CC (2003). Respiration-correlated spiral CT: A method of measuring respiratory-induced anatomic motion for radiation treatment planning. Med Phys.

[B31] Vedam SS, Keall PJ, Kini VR, Mostafavi H, Shukla HP, Mohan R (2003). Acquiring a four-dimensional computed tomography dataset using an external respiratory signal. Phys Med Biol.

[B32] Rietzel E, Pan T, Chen GTY (2005). Four-dimensional computed tomography: Image formation and clinical protocol. Med Phys.

[B33] Shirato H, Shimizu S, Kunieda T, Kitamura K, Kagei K, Nishioka T, Hashimoto S, Fujita K, Aoyama H (2000). Physical aspects of a real-time tumor-tracking system for gated radiotherapy. Int J Radiat Oncol.

[B34] Berbeco RI, Jiang SB, Sharp GC, Chen GTY, Mostafavi H, Shirato H (2004). Integrated radiotherapy imaging system (IRIS): design considerations of tumour tracking with linac gantry-mounted diagnostic x-ray systems with flat-panel detectors. Phys Med Biol.

[B35] Shirato H, Harada T, Harabayashi T, Hida K, Endo H, Kitamura K, Onimaru R, Yamazaki K, Kurauchi N, Shimizu T, Shinohara N, Matsushita M, aka-Akita H, Miyasaka K (2003). Feasibility of insertion/implantation of 2.0-mm-diameter gold internal fiducial markers for precise setup and real-time tumor tracking in radiotherapy. Int J Radiat Oncol Biol Phys.

[B36] Shimizu S, Shirato H, Kitamura K, Shinohara N, Harabayashi T, Tsukamoto T, Koyanagi T, Miyasaka K (2000). Use of an implanted marker and real-time tracking of the marker for the positioning of prostate and bladder cancers. Int J Radiat Oncol.

[B37] Schweikard A, Shiomi H, Adler J (2004). Respiration tracking in radiosurgery. Med Phys.

[B38] Rietzel E, Rosenthal SJ, Gierga DP, Willet CG, Chen GT (2004). Moving targets: detection and tracking of internal organ motion for treatment planning and patient set-up. Radiother Oncol.

[B39] Baroni G, Ferrigno G, Pedotti A (1998). Implementation and application of real-time motion analysis based on passive markers. Med Biol Eng Comput.

[B40] Kubo HD, Len PM, Minohara S, Mostafavi H (2000). Breathing-synchronized radiotherapy program at the University of California Davis Cancer Center. Med Phys.

[B41] Ford EC, Mageras GS, Yorke E, Rosenzweig KE, Wagman R, Ling CC (2002). Evaluation of respiratory movement during gated radiotherapy using film and electronic portal imaging. Int J Radiat Oncol.

[B42] Smith N, Meir I, Hale G, Howe R, Johnson L, Edwards P, Hawkes D, Bidmead M, Landau D (2003). Real-time 3D surface imaging for patient positioning in radiotherapy. Int J Radiat Oncol.

[B43] Kuechler S, Hoinkis C, Thierfelder C, Becker B, Zahn P, Geyer P, Lehmann D (2004). Respiratory motion – first investigations on the optimization of gating parameters. Int J Radiat Oncol.

[B44] Bert C, Metheany KG, Doppke K, Chen GT (2005). A phantom evaluation of a stereo-vision surface imaging system for radiotherapy patient setup. Med Phys.

[B45] Li XA, Stepaniak C, Gore E (2006). Technical and dosimetric aspects of respiratory gating using a pressure-sensor motion monitoring system. Med Phys.

[B46] Willoughby TR, Forbes AR, Buchholz D, Langen KM, Wagner TH, Zeidan OA, Kupelian PA, Meeks SL (2006). Evaluation of an infrared camera and X-ray system using implanted fiducials in patients with lung tumors for gated radiation therapy. Int J Radiat Oncol Biol Phys.

[B47] Rietzel E, Chen GTY, Choi NC, Willet CG (2005). Four-dimensional image-based treatment planning: Target volume segmentation and dose calculation in the presence of respiratory motion. Int J Radiat Oncol.

[B48] Bert C, Rietzel E (2007). 4D treatment planning for scanned ion beams. Radiat Oncol.

